# Complete Genome Sequence of Escherichia coli O157:H7 Phage UAE_MI-01, Isolated from Bird Feces

**DOI:** 10.1128/MRA.00348-21

**Published:** 2021-07-15

**Authors:** Mohamad Ismail Sultan-Alolama, Khaled A. El-Tarabily, Ranjit Vijayan

**Affiliations:** a Zayed Complex for Herbal Research and Traditional Medicine, Department of Health, Abu Dhabi, United Arab Emirates; b Department of Biology, College of Science, United Arab Emirates University, Al Ain, United Arab Emirates; Portland State University

## Abstract

Bacteriophage UAE_MI-01 is an Escherichia coli O157:H7 phage that was isolated from the feces of wild pigeons (*Columba livia domestica*) in Abu Dhabi, United Arab Emirates. All previously reported E. coli O157:H7 phages were isolated from ruminants. Here, we report the genetic features of this phage based on its complete genome sequence. UAE_MI-01 has the potential to be used as a therapeutic agent and as an industrial food preservative.

## ANNOUNCEMENT

Shiga toxin-producing Escherichia coli serotype O157:H7 is a common bacterium that causes foodborne illnesses (1–3). Applications of virulent phages as an alternative to antibacterial agents and as food preservatives are well established (4–6), and several of these products are now available commercially (7). There has also been an increase in phage-based therapy centers and clinical trials of therapeutic phage products (8).

One hundred grams of pigeon feces from under one nest (24.330293N, 54.618659E) was mixed with 250 ml of sterile water and seeded with 10^9^ CFU of O157:H7. After incubation at 37°C for 1 day with shaking, an additional 10^9^ CFU of O157:H7 was added. After 3 days, filtered supernatant (0.22 μm) was serially diluted in lambda buffer and screened for O157:H7 phages using the phage plaque assay (9). The largest and clearest plaque was isolated for further studies. Lytic activity was determined based on plaque morphology and the optical density at 600 nm (OD_600_) of the culture (9). The OD_600_ of the broth was reduced from 1.1 to 0.6 after 9 h of incubation of the O157:H7-phage mixture.

Phage DNA was extracted using a phage DNA isolation kit (Norgen Biotek Corp., Ontario, Canada) according to the manufacturer’s protocol. DNA library preparation and whole-genome sequencing were performed by Macrogen (South Korea). The DNA library was prepared using a TruSeq Nano DNA library preparation kit (Illumina, San Diego, CA, USA) and sequenced on an Illumina MiSeq sequencer. A total of 8,217,806 paired-end raw reads of 301-bp length were obtained. Quality checking was performed with FastQC v0.11.5 (https://www.bioinformatics.babraham.ac.uk/projects/fastqc). Adapters and low-quality reads were trimmed with minimum quality scores of Q30 using BBDuk v38.84. Reads less than 50 bp in length were discarded. Due to the large number of reads produced, the reads were randomly subsampled into five sets of 50,000 paired-end reads each (10); these were independently assembled *de novo*, using the Geneious assembler in Geneious Prime v2021.0.3 (https://www.geneious.com), and each produced the same circular contig. The online prokaryotic genome annotation service Rapid Annotation using Subsystem Technology (RAST) (https://rast.nmpdr.org) was used to identify open reading frames (ORFs), and proteins were annotated using BLASTP (https://blast.ncbi.nlm.nih.gov). The tRNAScan-SE search tool (http://lowelab.ucsc.edu/tRNAscan-SE/index.html) was used to search for tRNA genes. All tools were run with default parameters unless otherwise specified.

Phage UAE_MI-01 contained a circular phage genome of 44,281-bp length, with an average GC content of 54.7% and coverage of 235×. A BLASTN search indicated that it shared 92.6% sequence identity (98% coverage) with Escherichia phage SSL-2009a (GenBank accession number FJ750948) and 92.8% sequence identity (96% coverage) with Escherichia phage YD-2008.s (GenBank accession number KM896878). The phage was predicted to possess 64 ORFs and no tRNA genes. Its genome contained predicted structural/assembly genes, including those for terminase, HNH endonuclease, capsid protein, major and minor tail proteins, tail assembly proteins, and tape measure proteins. Replication/transcription-related genes included those for DNA polymerase I, helicase, and helicase-primase. The presence of endolysin and holin-like genes suggests that the lytic phage employs this mechanism for disruption of host cell membranes.

### Data availability.

The genome sequence of the phage UAE_MI-01 has been deposited in GenBank under the accession number MW862804. The sequencing reads have been deposited under the accession number PRJNA719367. The version of the phage genome described in this paper is the first version.
